# How the Context Matters. Literal and Figurative Meaning in the Embodied Language Paradigm

**DOI:** 10.1371/journal.pone.0115381

**Published:** 2014-12-22

**Authors:** Valentina Cuccio, Marianna Ambrosecchia, Francesca Ferri, Marco Carapezza, Franco Lo Piparo, Leonardo Fogassi, Vittorio Gallese

**Affiliations:** 1 Department of Neuroscience, University of Parma, Parma, Italy; 2 Department of Humanities, University of Palermo, Palermo, Italy; 3 Mind, Brain Imaging and Neuroethics, University of Ottawa, Institute of Mental Health Research, Ottawa, ON, Canada; UCLA, United States of America

## Abstract

The involvement of the sensorimotor system in language understanding has been widely demonstrated. However, the role of context in these studies has only recently started to be addressed. Though words are bearers of a semantic potential, meaning is the product of a pragmatic process. It needs to be situated in a context to be disambiguated. The aim of this study was to test the hypothesis that embodied simulation occurring during linguistic processing is contextually modulated to the extent that the same sentence, depending on the context of utterance, leads to the activation of different effector-specific brain motor areas. In order to test this hypothesis, we asked subjects to give a motor response with the hand or the foot to the presentation of ambiguous idioms containing action-related words when these are preceded by context sentences. The results directly support our hypothesis only in relation to the comprehension of hand-related action sentences.

## Introduction

In the last years many empirical findings have shown the involvement of the sensorimotor system in language understanding [1]–[3]. Listening to a sentence such as “John grasps the glass” determines the activation of hand-related areas of the motor cortex even if we are not carrying out any hand-related action. These findings suggest that linguistic meaning is grounded in systems for action and perception and directly challenge the idea that human cognitive abilities can be described in terms of operations on amodal and abstract symbols proposed by classic cognitivists [4], [5].

In this regard, behavioural studies revealed a facilitation effect between the processing of action-related words or sentences and the performance of concurrent and compatible motor acts [6]–[9]. An interference effect has also been observed in another set of studies (e.g. [8], [10]–[13]). The occurrence of either facilitation or interference is thought to depend on the extent of the temporal overlapping between linguistic and motor tasks [10], [12], [14]. Furthermore, neuroimaging [15]–[18], and neurophysiological studies, the latter realized by means of Transcranic Magnetic Stimulation (TMS) [8], [13], [19], [20], have widely revealed activation of the motor cortex when participants of the studies read or listened to action-related language. These studies showed that motor activations elicited by processing of action words and sentences follow a somatotopic organization.

Interestingly, somatotopic motor activation has been observed also during the comprehension of abstract and figurative use of language such as metaphors and idioms [21]–[25]. However, these latter findings are still controversial and challenging results have been reported in other set of studies [26], [27].

Recently, several review articles have been devoted to the task of critically analyzing the ever-increasing amount of behavioural, neuroimaging and neurophysiological data on this topic [28]–[31]. Among others, the most debated issues are currently those regarding the constitutive and automatic involvement of the sensorimotor system in language understanding. In other words, the hypothesis that the activation of the sensorimotor system is always a *necessary* condition to comprehend language, at least when language refers to the domains of action and perception, and whether this activation is sensible to factors such as contextual cues, is under investigation. This work will focus precisely on this latter topic, namely the role of context in modulating motor simulation.

It is worth noting here that authors working in embodied language research adopt an operative definition of semantics, described as a set of fixed relations between linguistic signs and some forms of knowledge. Language is conceived as a rigid code where words, meanings and–in the light of neuroscientific evidence–patterns of neural activations are fixedly associated. There are two hypotheses on which such an idea of semantics rests: 1) The compositional nature of meaning allowing to derive the meaning of a sentence from that of its word elements [32], [33]; 2) The fact that a linguistic expression could have meaning in the absence of a context. This really seems very doubtful, though points to an important intuition: words, though they can be modulated, are bearers of a semantic potential, which however in the absence of a context remains extremely vague.

The notion of context deserves an explanation. There is a propositional context, i.e. the expression in which the term occurs [34]. Then there is a broader context, the co-textual one. This means that the meaning of an expression is connected to the co-text, i.e. to the expressions that precede and follow it in a text [35]. There is a further meaning of context which is also essential, the pragmatic background, i.e. what the speaker does using a certain expression, the nonverbal context in which the speech act takes place [36], or the linguistic game, to use a Wittgensteinian term [37].

The pragmatic context is more than a mere element of enrichment of the linguistic expression; in fact, it is a fundamental feature of its possibility to signify something. In this view, meaning is always conceived of as the product of a pragmatic process and needs to be situated in a context to be correctly disambiguated and understood.

While the role of context in modulating embodied simulation during action recognition has already been demonstrated and widely discussed [38]–[41], only recently has this issue been addressed in embodied language research [25], [42]–[45]. What seems to still be missing is a redefinition of the ideas of meaning and semantics based on the notion of usage. Meaning, in this view, is a dynamic process in which both speaker and hearer are actively involved. Until now, when contextual effects have been taken into account, they have been conceived of as something given outside of speakers and that interacts with prefixed meanings in their “heads”. In the light of the definition of meaning as usage, we can instead think of meaning as the product of a process that entails different mechanisms such as, motor simulation and the integration of co-textual and contextual information. Significantly, this definition of meaning allows us to hypothesize that the mechanism of embodied simulation [46], [47] is part of the process of language comprehension in spite of being an automatic mechanism and is sensible to the context, considering context in its broadest sense.

## Aim of the study

The aim of the study is to test the hypothesis that embodied simulation occurring during linguistic processing can be contextually modulated to the extent that the very same sentence, depending on the context of utterance, leads to the activation of different effector-specific areas of the brain. Two previous studies have shown that the same target sentence, depending on the context of utterance, can activate in different ways the motor system [45], [48]. In a fMRI study, van Ackeren and colleagues [45] looked at motor simulation during the comprehension of indirect speech acts. Authors of this study showed that the comprehension of indirect requests, for example the sentence “It is hot here” uttered in a room with a window and interpreted as a request to open the window, activates the motor system much more reliably than the comprehension of the same sentence uttered in a different context, e.g. in the desert, and interpreted as a statement. Egorova, Shtyrov and Pulvermüller [48], in a study carried out with the time-resolved event-related potential (ERP) technique, also looked at different types of speech acts realized by means of the same target sentence. In this study, a critical word, considered as the target-sentence, was preceded by two different context sentences. According to the context of utterance, the same word was used to name or to request an object. Egorova and colleagues found that request-evoked potentials were larger in amplitude than those for naming. Significantly, the fronto-central cortex was the source underlying the ERP enhancement for Request suggesting the activation of motor knowledge. As far as we know, no study on figurative language has ever evaluated the role of pragmatic context on motor simulation in a similar way. This is precisely what we are going to do in this study. Previous works have mainly looked at motor simulation elicited by an action-related verb when this verb appears in two different, although similar, sentences, one literal and one figurative (for example, “Pablo kicks the ball” vs “Pablo kicks the habit”). In our study, we are going to look at motor simulation elicited by the very same target sentence when preceded by two different contexts (for example, the sentence “Pablo cuts the rope” preceded by two different sentences). We hypothesize that, according to the context of utterance, the very same sentence can recruit in different ways the motor system. In order to test this hypothesis, we assessed embodied simulation occurring during the comprehension of idiomatic and literal sentences containing action-related words when these sentences are preceded by a brief context sentence.

Idioms are multi-word units whose meaning does not correspond to the sum of their component parts [49]. They are considered conventional by definition. In fact, as Numberg, Sag and Wason [50] suggested, apart from the property of conventionality, none of the property usually ascribed to idioms (e.g. inflexibility, figuration, proverbiality, etc…) applies obligatorily to all idioms. In our study, we selected a list of Italian ambiguous idioms containing an action-related word. An idiom is defined as ambiguous if it has a plausible meaning both in the literal and in the figurative usage (ex. To kick the bucket). For each idiom (target sentence) we worked out two brief context sentences that can trigger respectively the literal or the figurative interpretation of the target sentence. Stimuli were constructed in such a way that when idiomatic meanings were elicited, verbs considered as arm/hand-action related described leg/foot actions and vice versa.

Following Borregine and Kaschak [7], a facilitation effect is expected only under conditions in which the same features are simultaneously active for different tasks (e.g., linguistic and motor task). However, if one of the two different tasks is completed (for example, if a full simulation of the action-related word has run), then the common features will be temporarily bound to that task (linguistic processing in this example) becoming unavailable to the other task (the motor act). In the light of this, in our study a facilitation effect is expected during the processing of literal sentences when participants respond with the congruent effector. In this case, they likely have enough (but not all) information to perform the motor task at an earlier stage of linguistic processing, while they have to wait until the end of the sentence in the case of idiomatic meanings. It is worth noting that idioms are considered as semantically transparent when there is a direct connection between the phrase and its figurative meaning, that is, when the idiomatic meaning can be guessed from the meaning of the words forming the phrase; when no such direct connection is available and the relationship between the phrase and the figurative meaning is arbitrary, idioms are considered as opaque. Considering that transparent idioms are supposed to be more easily imageable and that their imageability can affect embodied representations [51]–[54], we decided to select only transparent idioms. In this way, the role of context in modulating motor simulation is even more evident. In fact, although transparent idioms, due to the process of mental imagery, could trigger a motor simulation related to their literal meaning, we aimed to show that contextually-based interpretation prevails over literal meaning interpretation.

Furthermore, a questionnaire was also administered to exclude that our results could be explained by the degree of imageability of each item. Results from the questionnaire ruled out this possibility.

### Validation of the stimuli

We selected 20 Italian ambiguous idioms. Each idiom contained a verb or a noun relating to an action. For each idiom (target-sentence) we elaborated two brief sentences describing two different contexts (context sentences), in which the action could occur. Thus, we had 20 stimulus pairs. One context sentence in a stimulus pair elicited a literal interpretation of the target-sentence, whereas the other elicited an idiomatic interpretation of the same target-sentence. Each stimulus pair was constructed in such a way that when idiomatic meanings were elicited, verbs literally considered as arm-/hand- action related will describe leg-/foot- actions. Vice versa, verbs literally considered as leg-/foot- action related will describe arm-/hand-actions in the idiomatic meaning (see Table 1 and 2).

**Table 1:** Validated Stimuli.

Interpretation C+T	Context-sentences	Target-sentences	Evoked effector	T	F	A
Idiomatic	Il ladro si accorse che l'allarme risuonava nell'edificio	Tagliò la corda	foot	3	4	80%
Literal	Il marinaio si accorse che l'ancora si era incagliata al fondale		hand			85%
Idiomatic	L'automobilista spinse sull' acceleratore per arrivare in tempo	Calcò la mano	foot	4	4	87%
Literal	L'uomo esercitò una forte pressione sul coperchio per chiudere la scatola		hand			96%
*Idiomatic*	Voleva che la moglie non lo vedesse lì	Levò le tende	foot	3	4	75%
*Literal*	Voleva che la stanza non fosse così buia		hand			57%
*Idiomatic*	Decise di rimanere in piedi davanti a quella porta	Vi piantò le tende	foot	4	4	80%
*Literal*	Decise di accamparsi in quelle radura per la notte		hand			67%
Idiomatic	Il calciatore stava ancora provando lo stesso tiro in porta	Continuava a battere sullo stesso tasto	foot	4	3	92%
Literal	Il tecnico stava ancora aggiustando la lettera «L» della tastiera		hand			95%
Idiomatic	Aveva bisogno di una partner per la gara di ballo e lei si offrì	Gli diede una mano	foot	4	5	70%
Literal	Aveva bisogno di un appoggio per sollevarsi da terra e lui glielo offrì		hand			87%
Idiomatic	Stava percorrendo gli ultimi metri verso il traguardo	Era a portata di mano	foot	4	4	86%
Literal	Stava allungando il braccio verso la tazza sul tavolo		hand			93%
*Idiomatic*	Se fosse passata davanti alla sua scrivania lui l'avrebbe seguita	Gettò l'amo	foot	3	4	59%
*Literal*	Se avesse pescato da quel pontile avrebbe preso molti pesci		hand			95%
Idiomatic	L'atleta stava superando il suo record nella corsa e non volle fermarsi	Doveva battere il ferro finchè era caldo	foot	4	4	86%
Literal	Il fabbro stava martellando su una lastra incandescente e non volle fermarsi		hand			94%
Idiomatic	C'era già buio e aveva ancora molti chilometri davanti a sé	Si rimboccò le maniche	foot	4	4	76%
Literal	C'era già caldo e indossava ancora una camicia di velluto		hand			99%
Idiomatic	Il nuovo contratto era pronto per la firma, dopo qualche incertezza si decise	Saltò il fosso	hand	3	3	75%
Literal	La strada era interrotta per una buca, dopo qualche incertezza si decise		foot			92%
Idiomatic	A cena c'erano delle pietanze pessime	Le aveva fatte con i piedi	hand	3	4	81%
Literal	Sulla sabbia c'erano delle impronte		foot			90%
*Idiomatic*	Se gli avesse strappato i documenti avrebbe avuto lui la promozione	Gli fece lo sgambetto	hand	4	3	55%
*Literal*	Se lo avesse fatto cadere avrebbe vinto lui la maratona		foot			98%
Idiomatic	Era il momento adatto per provare a scambiarsi una stretta di mano	Luca fece il primo passo	hand	4	4	90%
Literal	Era il momento adatto per provare a camminare da solo		foot			94%
Idiomatic	Se lei avesse firmato quell'accordo lui ci sarebbe rimasto male	Decise di pestargli i piedi	hand	4	4	70%
Literal	Se lui le avesse ancora fatto piedino lei avrebbe reagito		foot			97%
*Idiomatic*	Le sigarette erano proibite in quel locale	Paolo ci passò sopra	hand	3	4	67%
*Literal*	I documenti le erano caduti sul pavimento		foot			73%
*Idiomatic*	Il giocoliere si presentò al pubblico	Era arrivato per lui il momento di entrare in campo	hand	4	4	36%
*Literal*	Il calciatore giocò la sua prima partita		foot			92%
Idiomatic	Il compito di disegno era molto difficile	Si fermò quasi ad ogni passo	hand	3	3	87%
Literal	La strada verso il paese era piena di ostacoli		foot			95%
Idiomatic	Al pittore mancavano gli ultimi tocchi di colore sulla tela	Era ad un passo dal traguardo	hand	4	4	90%
Literal	All'atleta mancavano pochi centimetri all'arrivo		foot			97%
Idiomatic	Il chirurgo si trovava in una fase dell'operazione delicata e pericolosa	Stava camminando lungo una strada accidentata	hand	4	3	82%
Literal	L'escursionista si trovava in un tratto pieno di ostacoli e pericoloso		foot			91%

Subject ratings of stimuli, and selected stimuli for experiments 1 and 3. T = Transparency; F = Familiarity; A = Accuracy. Stimuli in italic were excluded.

**Table 2:** Validated Stimuli: English translation.

Interpretation C+T	Context-sentences	Target-sentences	Evoked effector	T	F	A
Idiomatic	The thief realized that the alarm was sounding aloud in the building	**He ran away**	foot	3	4	80%
Literal	The sailor realized that the anchor was stuck in the seabed	*He cut the rope*	hand			85%
Idiomatic	The driver hit the gas pedal to be there in time	**He exaggerated**	foot	4	4	87%
Literal	The man pressed the top of the box to close it	*He pressed the hand on it*	hand			96%
Idiomatic	He didn't want his wife to see him there	**He left**	foot	3	4	75%
Literal	He didn't want that room to be so dark	*He removed the curtains*	hand			57%
Idiomatic	He decided to stand in front of that door	**He set up camp there**	foot	4	4	80%
Literal	He decided to put up his tent there for the night	*He set up camp there*	hand			67%
Idiomatic	The football player was still practising the same shot at the goal	**He was insisting**	foot	4	3	92%
Literal	The technician was fixing the “L” key on the keyboard	*He kept beating on the same key*	hand			95%
Idiomatic	He needed a partner for the dance contest and she volunteered	**She gave him a hand**	foot	4	5	70%
Literal	He needed to support to get up from the ground and she offered	*She gave him a hand*	hand			87%
Idiomatic	He was running the last metres to the finish line	**It was within reach.**	foot	4	4	86%
Literal	He was extending out his arm towards the cup on the table	*It was within reach*	hand			93%
Idiomatic	If she had passed by his desk he would have followed her	**She had laid the bait**	foot	3	4	59%
Literal	If she had fished from that pier she would have caught a lot of fishes	*She had laid the bait*	hand			95%
Idiomatic	The athlete was breaking his running record and he did not want to stop	**He had to strike while the iron was hot**	foot	4	4	86%
Literal	The blacksmith was hammering a white-hot sheet of iron and he did not want to stop	*He had to strike while the iron was hot*	hand			94%
Idiomatic	It was already dark and he had still many kilometres to walk in front of him	**So he rolled up his sleeves**	foot	4	4	76%
Literal	It was already hot and I was wearing a long-sleeved shirt	So he rolled up his sleeves	hand			99%
Idiomatic	The new contract was ready to be signed, after some uncertainty he decided	**He signed it**	hand	3	3	75%
Literal	The road was interrupted because of a pit, after some uncertainty he decided	*He jumped over the moat*	foot			92%
Idiomatic	There were awful dishes at dinner	**She has done them with her feet**	hand	3	4	81%
Literal	There were tracks on the sand	*She has done them with her feet*	foot			90%
Idiomatic	If she ripped up his documents she would have the promotion instead of him	**She tripped him**	hand	4	3	55%
Literal	If he made him fall down he would win the marathon instead of him	*He tripped him*	foot			98%
Idiomatic	It was the right time to offer him a handshake	**Luca took the first step**	hand	4	4	90%
Literal	It was the right time to try to walk by himself	*Luca took the first step*	foot			94%
Idiomatic	She signed the agreement herself and that made him upset	**She stepped on his toes**	hand	4	4	70%
Literal	He rubbed his foot against her leg again, so she reacted	*She stepped on his toes*	foot			97%
Idiomatic	The sign said that smoking was not allowed in that place	**Paolo just walked all over it**	hand	3	4	67%
Literal	The document fell onto the floor	*Paolo just walked all over it*	foot			73%
Idiomatic	The juggler presented himself to the audience	**It was the time for him to start doing his games**	hand	4	4	36%
Literal	The football player played his first match	*It was the time for him to enter the field*	foot			92%
Idiomatic	The recipe was very difficult	**He stopped at almost every single step**	hand	3	3	87%
Literal	The path that he took to the village was full of obstacles	*He stopped at almost every single step*	foot			95%
Idiomatic	The painter needed to do the last brush strokes on the painting	**The finish line was in sight**	hand	4	4	90%
Literal	The athlete needed to do the last steps towards the goal	*The finish line was in sight*	foot			97%
Idiomatic	The surgeon was at a difficult and dangerous stage of the surgery	**He was taking a treacherous road**	hand	4	3	82%
Literal	The hiker was on a difficult and dangerous path	***He was taking a treacherous road***	foot			91%

English translation of stimuli. T = Transparency; F = Familiarity; A = Accuracy.

The literal translation of idioms from one language to another is not always possible because not always there is a direct correspondence between idiomatic expressions of different languages. Because the idioms in our experiment are ambiguous, their literal translation is always possible when they are used as literal sentences. However, when they are used as figurative sentences, is not always possible to translate them word by word. For this reason, when a correspondent idiom was not found in English, we decided to translate idioms in figurative sentences according to their inferential meaning. Target sentences in italic are the literal translations of Italian ambiguous idioms used as literal sentences. Target sentences in bold are the translations of Italian ambiguous idioms used as idiomatic sentences.

Italian stimuli where balanced on the basis of number of syllables, grammatical structure, verbal time, length of the sentence. This was not always possible in the English translation.

In summary, the same target-sentences could appear two times in each block, but each time it was preceded by a different context-sentence eliciting a different interpretation of its meaning.

### Participants

Forty right-handed students (mean age 25 years, range 19–32) entered the validation phase. They were recruited at the University of Parma. All of them were native Italian speakers and reported no history of speaking disorders. They had normal or corrected-to-normal vision. None of them was aware of the purpose of the experiment. The experimental protocols of the validation and of the three experiments were approved by the Ethics Committee of the University of Parma (Comitato Etico Provinciale – AUSL di Parma) and all participants of the experiments gave written informed consent. Experiments were conducted in accordance with the ethical standards of the 1964 Declaration of Helsinki.

### Experimental procedure

Stimuli were presented at the centre of a computer screen on a white background. They were written in black lowercase, with Arial font. Participants were presented with 120 trials grouped in 3 blocks. Each stimulus was presented once in a block. Each trial started by presenting a fixation cross at the centre of the screen for 800 ms. Then, a context-sentence was presented for 4000 ms. Finally, a target-sentence was presented for a maximum duration of 3000 ms. The trial stopped when participants pressed a response-key. Stimuli appeared in random order.

Participants had to explicitly judge whether the target-sentence described a hand- or a foot- related action based on what they could know from the preceding context sentence. They were instructed to respond by pressing a left or a right previously assigned response key, with their right index finger.

The response keys were counterbalanced between subjects. Each participant was also required to rate each idiom on two rating scales, one for the idiom familiarity (i.e., how much the idiom meaning was known) and one for semantic transparency (i.e., how much the meaning of the words composing each idiom string contributed to the figurative meaning; [55]. In both cases, participants rated each idiom by choosing a number from the 5-point Likert Scale (familiarity rating: 1 =  not at all, 5 =  very much; transparency rating; 1 = not at all, 5 =  very much). Idioms appeared in random order.

### Results

Four participants were removed from the analysis because their error rate exceeded 50% (wrong responses, missing responses). Results for each type of stimulus are reported in table 1. Experimental stimuli were selected according to the following criteria. Accuracy, ≥70%; familiarity, mean score ≥3; transparency, mean score ≥3 [56]. We selected only stimuli satisfying all these three criteria. Accordingly, 6 target-sentences and the associated context sentences were discarded from the list, because they didn't match established criteria (see table 1 and 2).

Ratings given on the accuracy, familiarity and transparency of idioms containing a hand action verb do not differ significantly from those containing a foot action verb (Accuracy: hand target-sentences, mean  = 82.99%, SE = 0.02 vs foot target-sentences, mean = 82.65%, SE = 0.03, t6 = −0.199, p = 0.848; transparency: hand target-sentences, mean  = 3.70 SE = 0.08, vs foot target-sentences, mean = 3.67, SE = 0.05; t_9_ = 0.142 p = 0.15; familiarity: hand target-sentences, mean = 4.04 SE = 0.08, vs foot target-sentences, mean = 3.66, SE = 0.07; t_9_ = 1.720, p = 0.119).

The validation of the stimuli allowed us to select only those pairs of sentences in which participants drew the expected inference in the interpretation of the target.

## Experiment 1

We set this experiment to test if embodied simulation occurring during linguistic processing can be contextually modulated to the extent that the very same sentence, depending on the context of utterance, leads to the activation of different effector-specific areas of the brain.

### Participants

Twenty-two right-handed students (mean age 24.5 years, range 19–32) were recruited at the University of Parma to enter experiment 1. All of them were native Italian speakers and reported no history of speech disorders. None of them was aware of the purpose of the experiment. The study was approved by the local ethical committee.

### Procedure

The experiment was carried out in a sound-attenuated and dimly illuminated room. Participants sat comfortably in front of a computer screen at a distance of about 60 cm from it. Linguistic stimuli consisting of 14 Italian ambiguous idioms (see table 1 and 2) were presented in the centre of the computer screen on a white background. They were written in black lowercase using Arial font. The presentation of the stimuli and the recording of the participants' responses were controlled by E-Prime software (Psychology Software Tools, Inc., Sharpsburg, PA). During the experiment each pair of context/target sentences was presented twice. Each of the 14 target-sentences was associated to three different context sentences: 1. Contexts that trigger the literal interpretation of the target; 2. Contexts that trigger the idiomatic interpretation of the target; 3. Contexts unrelated to the target interpretation (e.g. Context: “The Earth is part of the solar system and revolves around the Sun”, target: “He gave him an hand”).

The experiment consisted of 84 trials. We had 4 experimental conditions for the go-trials:

targets containing an arm/hand related word literally interpreted (Literal Hand; LH); targets containing an arm/hand related word idiomatically interpreted as describing a leg/foot action (Idiomatic Foot; IF); targets containing a leg/foot related word literally interpreted (Literal Foot; LF); targets containing a leg/foot related word idiomatically interpreted as describing an arm/hand action (Idiomatic Hand; IH).

Stimuli appeared in random order. Twelve trials before the Experiment served to familiarize with the task. Participants had to perform 120 trials grouped in 3 blocks. Each stimulus was presented once in a block. Each trial started by presenting a fixation cross at the centre of the screen for 800 ms.

Then, a context-sentence was presented for 4000 ms. Finally, a target-sentence was presented for a maximum duration of 3000 ms. The trial stopped when participants pressed a response-key.

Participants were instructed to carefully read the sentences and to press a red button on a keyboard with their right hand, as fast and accurately as possible, when they judged that there was a relation between the target-sentence and the context-sentence. They had to refrain from responding when they judged that there was no relation of relevancy between the target-sentence and the context-sentence (go-no go paradigm).

Two linear mixed-effects models were separately carried out on mean reaction times (RTs) and Analysis of correct responses (accuracy), with Interpretation of meaning (Idiomatic vs. Literal) and Effector of action (Hand vs. Foot) as fixed factors, and Items as random factors. We adopted the linear mixed effect model because it is a robust analysis that allows to control for the variability of items and subjects [57]. This type of analysis is widely used in language research because it prevents either the potential lack of power and the loss of information due to the prior averaging of the by-subject and by-item analyses [57], [58].

Significant differences were explored using Sidak's correction for multiple comparisons.

### Results

Two participants were excluded from the analysis as outliers (2.5 SD from the mean of the group). The mean percentage of correct response was 87.8% (SE = 0.03). Error trials were excluded from further analyses. The sum of correct response and the mean RTs were calculated for each condition; responses either longer or shorter than 2 standard deviations from the individual mean were treated as outliers and not considered (2% of the data set). Based on a further items analysis, we excluded 3 target-sentences and their relative context-sentences from the analysis, because they did not reach the 70% of correct responses “Continuava a battere sullo stesso tasto”/“He kept beating on the same key”, mean  = 67.5%, SE = 7.89 “saltò il fosso”/“He jumped over the moat”, mean = 68.7%, SE = 6.91 “Si fermò quasi ad ogni passo”/“He stopped at almost every single step”, mean = 66.2%, SE = 8.41). We excluded another item (“Tagliò la corda”/He cut the rope) because a deeper examination of the stimuli revealed that this was the only case in which the inferential meaning of the figurative sentence was stable (“To cut the rope” is always interpreted as meaning “To run away”) compared to the inferential meaning elicited by the other idioms that is more contextually determined. Also, the same items were not included in the next experiments.

Analysis of accuracy revealed only a significant main effect of Interpretation of Meaning (F_1,377_ = 24.22; p = 0.000001), as the interpretation of Idiomatic trials was more difficult than the interpretation of Literal trials (mean  = 81%, SE = 0.03 vs. mean = 90% SE = 0.06 of correct responses, respectively).

**Figure 1:**
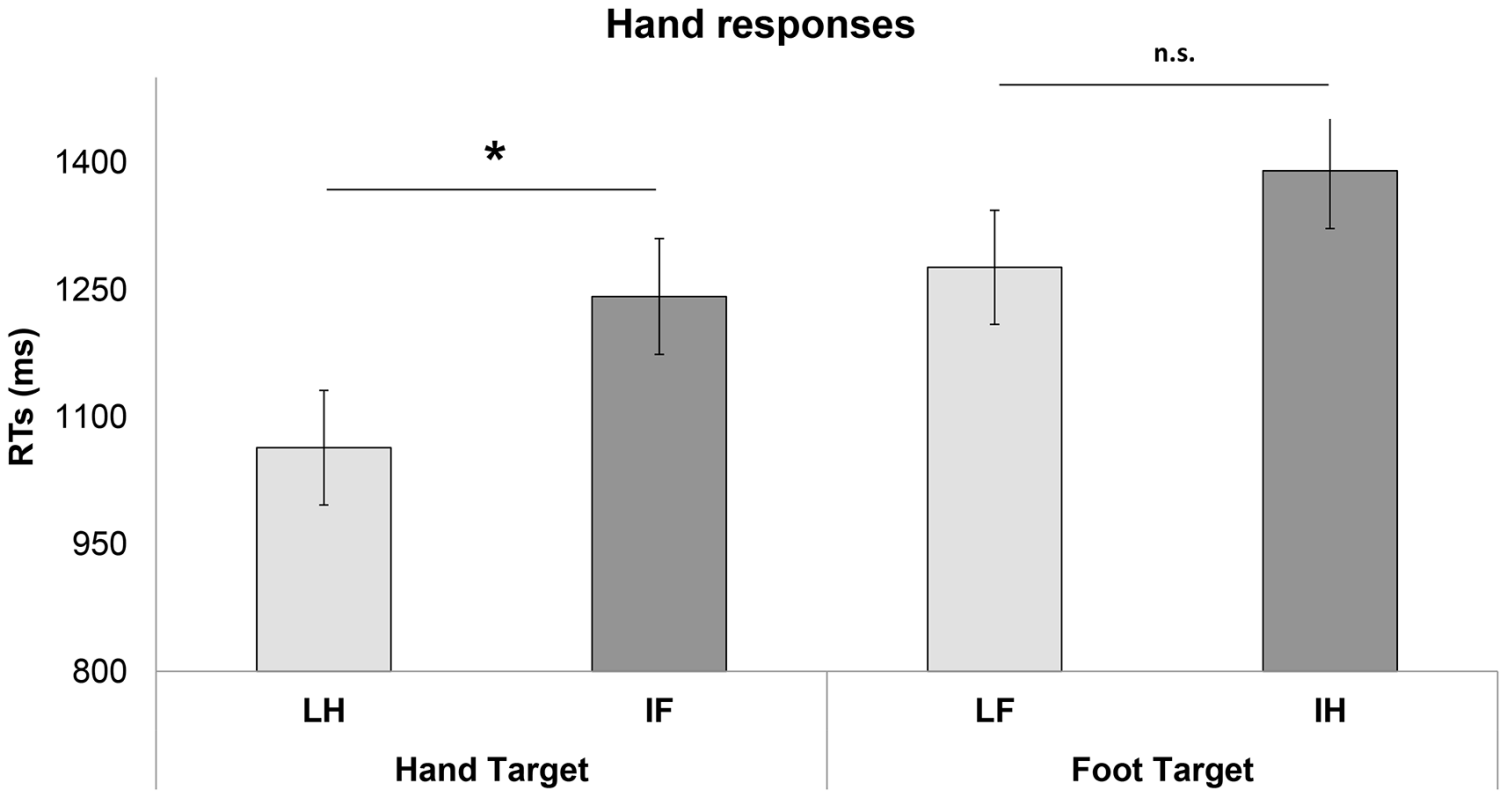
Experiment 1, hand responses. Mean RTs for hand/arm and leg/foot target-sentences. Vertical bars on the histograms indicate standard error of mean. The asterisk indicates a statistical significance between the means.

Analysis of RTs showed the main effect of Interpretation of meaning (F_1, 358_ = 19.16; p = 0,00001) showing that RTs to Literal trials were faster (mean  = 1171 ms; SE = 63.33) than idiomatic trials (mean 1315 ms; SE = 63.70). The interaction between Interpretation of Meaning and Effector of action was also significant (F_1, 358_ = 29.31; p = 0.0000001) with faster RTs in LH condition than IH condition (LH: mean  = 1064 ms, SE = 97.49; IH: mean = 1390, SE = 67.93; p = 0.000001). LF condition and IF condition did not differ from each other (LF: mean 1276 ms SE = 67.32; IF: mean = 1242 ms SE = 68.23.79 p = 0.97; see fig. 1).

In addition, RTs in LH condition were faster than in IF condition (LH: mean  = 1064 ms, SE = 67.49 IF: mean  = 1241 ms SE = 68.23; p = 0.001), while this was not the case for foot- related target-sentences.

(LF mean 1276 ms SE = 67.32; IH condition mean = 1242 SE = 68.23; p = 0.1).

This latter result can be interpreted as the product of a facilitation effect due to the interpretation of target sentences in specific contexts. However, it could also be objected that this facilitation effect, rather than being generated by the combination of target and context sentences, could be primarily determined by context-sentences, because these sentences contained action-related words that could induce a pre-activation in the motor system. Experiment 2 was set to rule out this possibility.

## Experiment 2

### Participants

Fifteen right-handed students (mean age 23.5, range 19–29 years) were recruited at the University of Parma for this experiment. All of them were native Italian speakers and reported no history of speech disorders. None of them was aware of the purpose of the experiment. The study was approved by the local ethical committee.

### Procedure

In this experiment stimuli and procedure were the same as in Experiment 1 except for target-sentences (see table 3). Each target sentence was replaced by an abstract sentence balanced on the basis of its syntactic structure, number of words and syllables, times and modes of the verb.

**Table 3:** Stimuli used in Experiment 2.

Context Activation	Context sentences	Abstract Target
**Foot**	L'automobilista spinse sull' acceleratore per arrivare in tempo	Rischiò la vita
	*The driver hit the gas pedal to be there in time*	*He risked his life*
**Hand**	L'uomo esercitò una forte pressione sul coperchio per chiudere la scatola	Sfogò la rabbia
	*The man pressed the top of the box to close it*	*He let of steam*
**Foot**	Aveva bisogno di una partner per la gara di ballo e lei si offrì	Le sembrò molto gentile
	*He needed a partner for the dance contest and she volunteered*	*He seemed very gentle to her*
**Hand**	Aveva bisogno di un appoggio per sollevarsi da terra e lui glielo offrì	Gli fece molta pena
	*He needed to support to get up from the ground and she offered*	*She felt so sorry for him*
**Foot**	Stava percorrendo gli ultimi metri verso il traguardo	Era fiero di sé stesso
	*He was running the last meters to the finish line*	*He was proud of himself*
**Hand**	Stava allungando il braccio verso la tazza sul tavolo	Era un momento di relax
	*He was reaching out his arm towards the cup on the table*	*It was a relaxing moment*
**Foot**	L'atleta stava superando il suo record nella corsa e non volle fermarsi	Dimostrava di sperare nel proprio successo
	*The athlete was breaking his running record and he did not want to stop*	*He showed to believe in his success*
**Hand**	Il fabbro stava martellando su una lastra incandescente e non volle fermarsi	Dimostrava di essere un vero perfezionista
	*The blacksmith was hammering a white-hot sheet of iron and he did not want to stop*	*He showed to be a perfectionist*
**Foot**	C'era già buio e aveva ancora molti chilometri davanti a sé	Si augurò buona fortuna
	*It was already dark and he had still many kilometres to walk in font of him*	*He wished for luck*
**Hand**	C'era già caldo e indossava ancora una camicia di velluto	Si sentì in imbarazzo
	*It was already hot and he was wearing a long-sleeved shirt*	*He felt embarrassed*
**Foot**	Sulla sabbia c'erano delle impronte	Lo aveva intrigato il mistero
	*There were tracks on the sand*	*He was intrigued by the mystery*
**Hand**	A cena c'erano delle pietanze pessime	Lo aveva preso il disgusto
	*There were awful dishes at dinner*	*He felt discussed*
**Foot**	Era il momento adatto per provare a camminare da solo	Luca provò una grande gioia
	*It was the right time to walk by himself*	*Luca felt a sense of joy*
**Hand**	Era il momento adatto per provare a scambiarsi una stretta di mano	Luca Provò una grande pace
	*It was the right time to offer him a handshake*	*Luca felt a sense of piece*
**Foot**	Se lui le avesse ancora fatto piedino lei avrebbe reagito	Pensò di incutergli timore
	*He rubbed his foot against her leg again, so she reacted*	*She thought to frighten him*
**Hand**	Se lei avesse firmato quell'accordo lui ci sarebbe rimasto male	Decise di mostrarsi di carattere
	*She signed the agreement herself and that made him upset.*	*She decided to show his strong character*
**Foot**	All'atleta mancavano pochi centimetri all'arrivo	Era il suo momento di gloria
	*The athlete needed to do the last steps towards the goal*	*It was his moment of glory*
**Hand**	Al pittore mancavano gli ultimi tocchi di colore sulla tela	Era soddisfatto di sé stesso
	*The painter needed to do the last brush strokes on the painting*	*He was pleased with himself*
**Foot**	L'escursionista si trovava in un tratto pieno di ostacoli e pericoloso	Stava riflettendo sulle probabilità di riuscita
	*The hiker was on a difficult and dangerous path*	*He was thinking about the chances of success*
**Hand**	Il chirurgo si trovava in una fase dell'operazione delicata e pericolosa	Stava apprezzando il fascino delle sfide
	*The surgeon was at a difficult and dangerous stage of the surgery*	*He was enjoying the fascination of challenges*

Stimuli used are in italic and the corresponding English translations in bold. Note that target sentences in this case are abstract sentences that did not describe any hand or foot actions.

Abstract target sentences did not describe any hand or foot actions. Participants were instructed to carefully read the sentences and to press a red button on a keyboard, as fast and accurately as possible, when they judged that there was a relation between the abstract target-sentence and the context sentence. They had to refrain from responding when they judged that there was no relation of relevancy between the target-sentence and the context sentence (go-no go paradigm).

RTs and Accuracy entered in two separated linear mixed-models with Context- activation (Arm/Hand vs. Leg/Foot) as fixed factors, and Items as random factors.

### Results

Trials in which participants failed to respond correctly were excluded from the analysis of RTs (mean = 6.9%, SE = 0.02). The mean percentage of correct response was 85.68% (SE = 0.02). Error trials were excluded from further analyses. The sum of correct response and the mean RTs were calculated for each condition; responses either longer or shorter than 2 standard deviations from the individual mean were treated as outliers and not considered (7% of the data set).

A previous item analysis showed that every item reached at least 70% of correct responses.

Regarding Accuracy, the main effect of Context-activation was significant (F_1,26_ = 7.84; p = 0.01). Participants were significantly less accurate when they responded to context-sentences with arm/hand action verbs than context-sentences with leg/foot action verbs (mean  = 88.8% SE = 0.04 vs. Mean  = 97.4% SE = 0.04).

**Figure 2:**
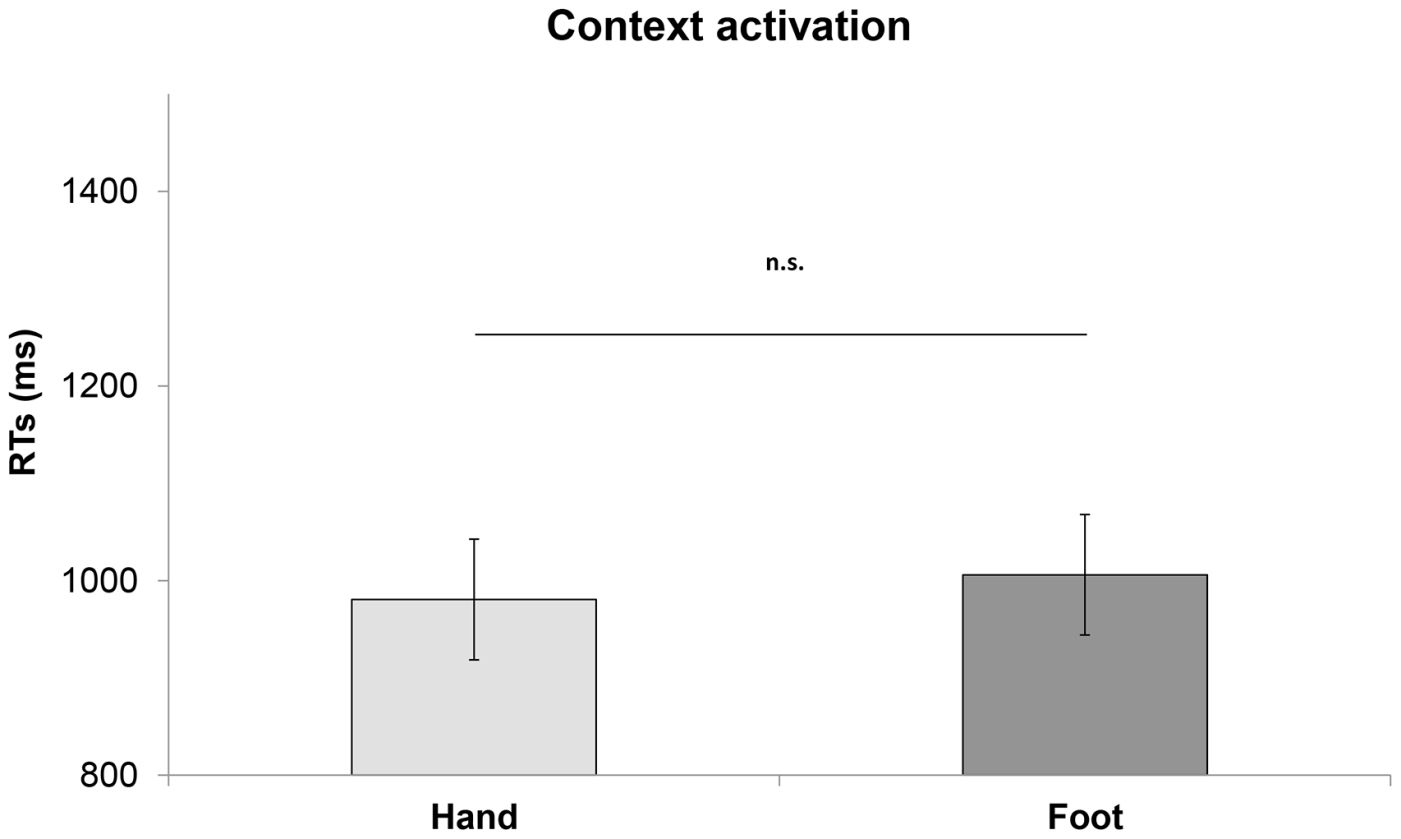
Experiment 2, hand responses. Mean RTs for context-sentences preceding abstract target-sentences. Vertical bars on the histograms indicate standard error of mean.

Analysis of RTs revealed that they do not differ between context-sentences of these two variables (F_1, 14.2_ = 0.50; p = 0.46; Arm/Hand context: mean  = 981, SE = 62.10 vs. Leg/Foot context mean  = 1005.63 ms SE = 61.87; see fig. 2) demonstrating that context sentences by themselves did not significantly facilitate the congruent responses (hand responses).

In Experiment 1, in which a hand response was requested, a literal hand facilitation effect was found. Analogously, a facilitation effect can be expected for the literal interpretation of target sentences containing foot-related words in comparison to the idiomatic (hand-related) interpretation of the same target sentences if a foot response is requested. Experiment 3 aimed at investigating this issue.

## Experiment 3

This experiment was carried out to verify whether a facilitation effect could be found during the literal interpretation of sentences containing foot-related words in comparison to the idiomatic (hand-related) interpretation of the same target sentences if participants responded with the foot. Furthermore, no significant difference in RTs was expected between the literal and idiomatic interpretation of sentences containing hand-related words.

### Participants

Twenty-three right-handed students (mean age 24.5 years, range 19–30) were recruited at the University of Parma for this experiment. They were native Italian speakers and reported no history of speech disorders. None of them was aware of the purpose of the experiment.

### Procedure

The experimental procedure was the same used in the Experiment 1 except for the fact that participants performed foot, rather than hand, responses on the same keyboard used in Experiment 1.

### Results

Three participants were discarded from the analysis because their error rates exceeded 30% (missing responses or wrong responses. Trials in which participants failed to respond correctly (3% of data set) were excluded from the analysis of RT). The mean percentage of correct response was 89.38% (SE = 0.03). Error trials were excluded from further analyses. The sum of correct responses and the mean RTs were calculated for each condition; responses higher or lower than 2 standard deviations from the individual mean were treated as outliers. An Item analysis showed that every item reached at least 60% of correct responses.

As in Experiment 1, two linear mixed-effects models were separately carried out on mean RTs and accuracy, with Interpretation of meaning (Idiomatic vs. Literal) and Effector of action (Hand vs. Foot) as fixed factor, and Items as random factors. Significant differences were explored using Sidak's correction for multiple comparisons.

Analysis of accuracy revealed only a significant main effect of Interpretation of Meaning (F_1, 377_ = 23.93; p<0. 01 as the interpretation of Idiomatic trials was more difficult than the interpretation of Literal trials (mean  = 83%, SE = 0.03 vs. mean = 95% SE = 0.07 of correct responses).

Analysis of RTs showed only a significant Interpretation of meaning by Effector of action significant interaction (F_1, 359_ = 11.49; p = 0.001;).

**Figure 3:**
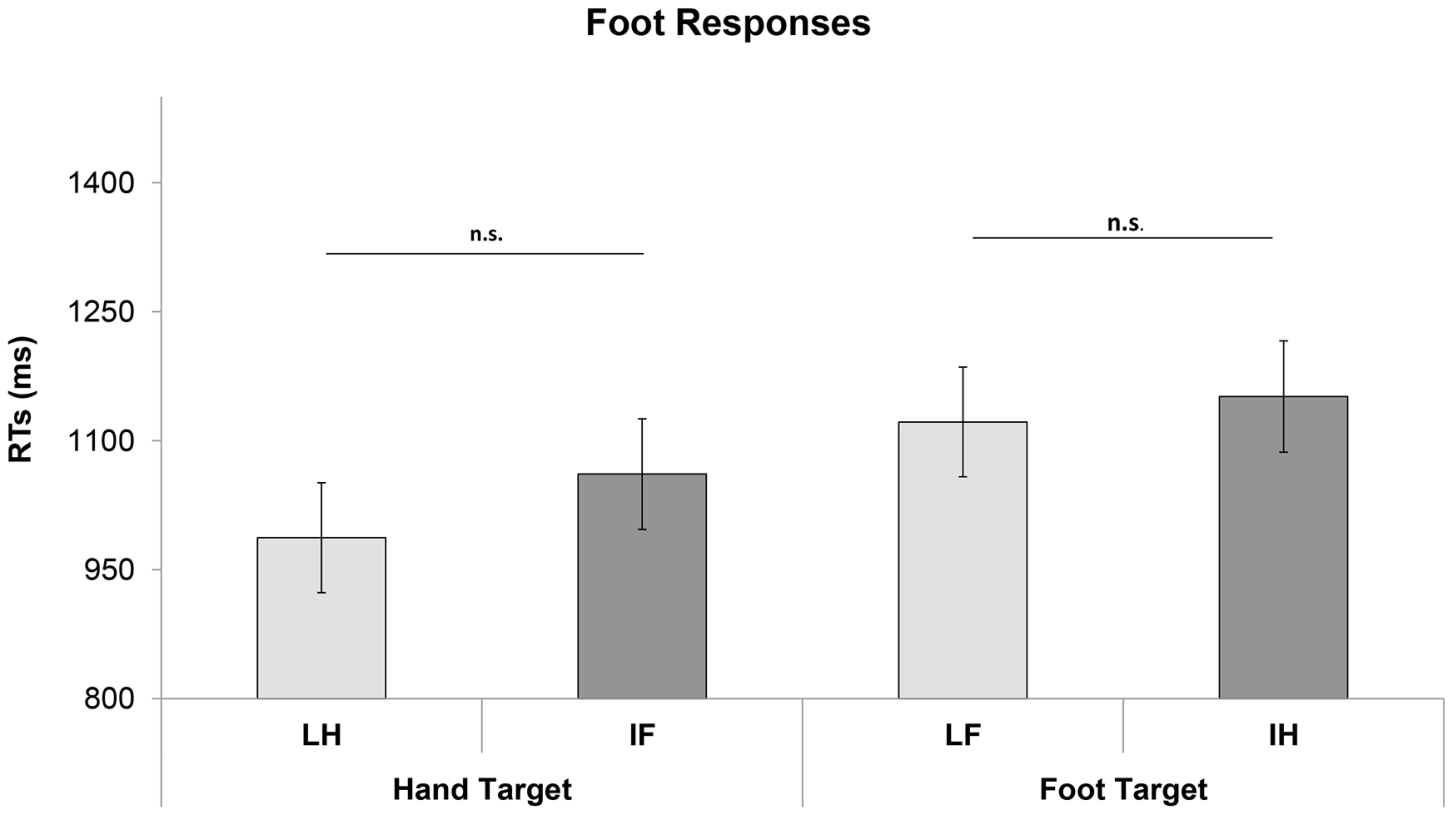
Experiment 3, foot responses. Mean RTs for hand/arm and leg/foot target-sentences. Vertical bars on histograms indicate standard error of mean. The asterisk indicates a statistical significance between the means.

Post hoc analyses (Sidak) revealed faster RTs in LH condition than IH condition (LH: mean  = 987 ms, SE = 64.01; IH condition, mean = 1151. SE = 64.67; p = 0.003). LF condition and IF condition did not differ from each other (LF: mean = 1121 ms, SE = 63.82; IF: mean = 1061 ms SE = 64.38; p = 0.72; see fig. 3). Differently from Experiment 1, post-hoc analysis revealed also that RTs for LH condition did not differ from RTs for IF condition (LH: mean  = 987 ms, SE = 64.01, IF: 1061 ms SE = 64.38; p = 0.52). LF and IH conditions, as in Experiment 1, did not differ significantly (LF: mean = 1121 ms, SE = 63.82; IH condition mean = 1151. SE = 64.67; p = 0.90).

### Imageability questionnaire

A further questionnaire was administered to exclude that our results could be explained by the degree of imageability of each item [51]–[54].

A new group of 38 right-handed students (mean age 26.02 years, range 19–35) was recruited following the same inclusion criteria previously described. They were asked to rate the imageability of each idiomatic/literal sentence presented in Experiment 1 and 3. For the rating we used a 101-point visual analogue scale (VAS), with 0 corresponding to *very little* and 100 corresponding to *very much*. Participants were required to evaluate how much they could imagine the action described in the sentence. The questionnaire was administered online using Qualtrics software, Version 37,892 of the Qualtrics Research Suite. Copyright 2014.

### Results

**Figure 4:**
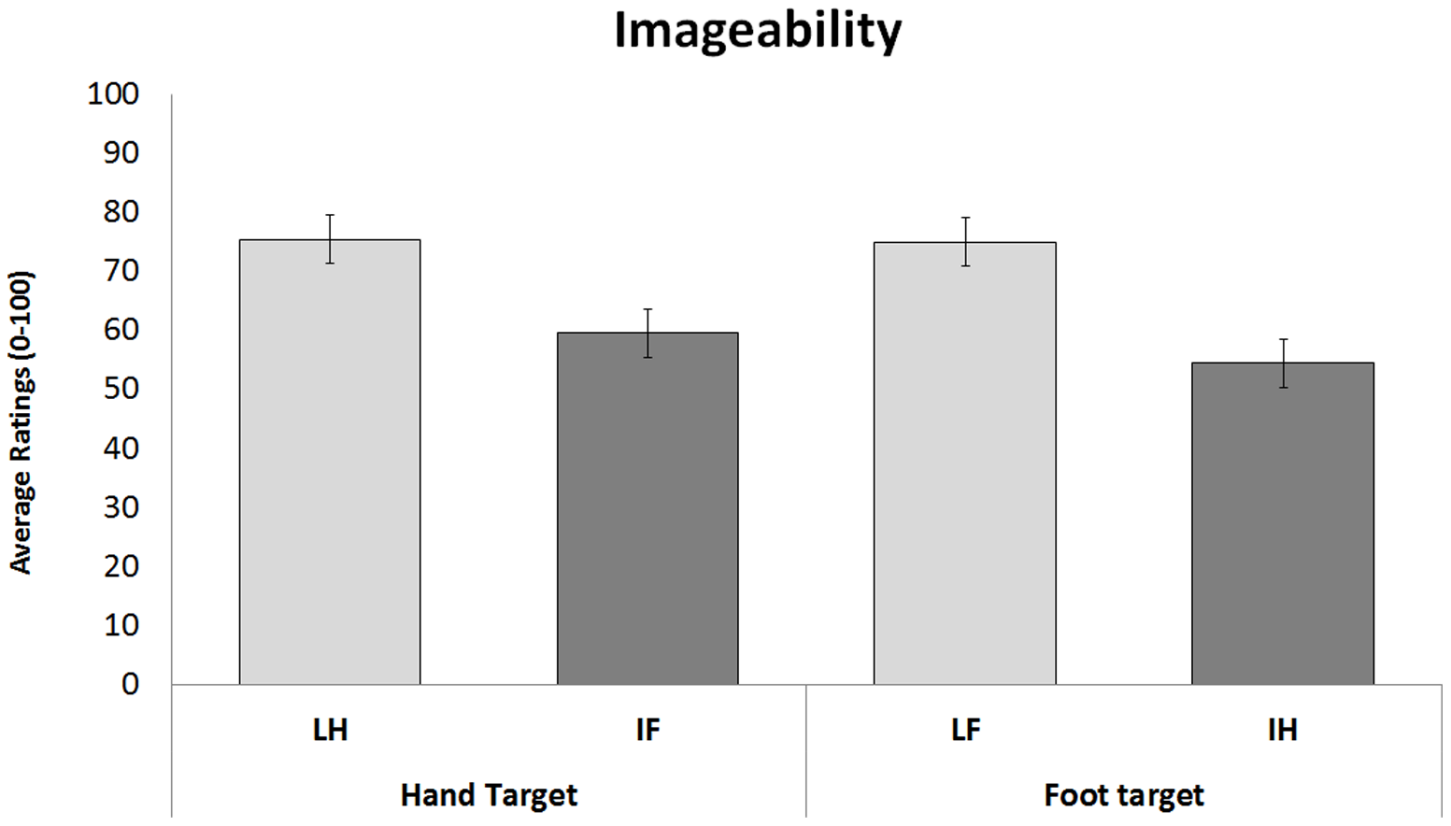
Imageability questionnaire. Mean ratings of participants for hand/arm and leg/foot target-sentences. Vertical bars on histograms indicate standard error of mean. The asterisk indicates a statistical significance between the means.

Ratings of participants were submitted to the same linear mixed-models used in Experiment 1 and 2. Interpretation of meaning (Idiomatic vs. Literal) and Effector of action (Hand vs. Foot) were fixed factors, whereas Items were random factors. In line with the results from Experiment 1 and 3, the analysis showed the main effect of Interpretation of meaning (F_1, 15.9_ = 19.38; p<0.01), as literal sentences were easier to imagine than idiomatic sentences (mean = 75.2/100 SE = 2.92 vs mean  = 57/100, SE = 2.93). However, neither the main effect of Effector (F_1, 15.9_ = 0.32 p = 0.58) nor the interaction between Meaning and Effector were significant (F_1, 15.9_ = 0.45 p = 0.51; see fig 4). These results clearly suggest that the degree of imageability alone could not fully explain the facilitation effect for the LH condition (faster RTs) compared to the IF and LF conditions observed in Experiment 1 (hand responses), but absent in Experiment 3 (foot responses).

## Discussion

This study aimed to test the hypothesis that motor simulation occurring during linguistic comprehension can be contextually modulated. In order to test this hypothesis we chose a list of 14 Italian ambiguous idioms containing action-related words and we predicted that the very same sentence activates in different ways the motor system according to its literal or idiomatic interpretation in the context of utterance. Our results directly support this hypothesis only in relation to the comprehension of hand-related action sentences.

In summary, each target sentence had two different possible interpretations with four factors at play: meaning (literal or idiomatic) and effector (hand or foot). Half of the target sentences contained a hand-related word and could be interpreted both as hand-related literal sentences and foot-related idiomatic sentences. The other half contained a foot-related word and could be interpreted both as foot-related literal sentences and hand-related idiomatic sentences.

When participants responded with the hand (Exp. 1), we observed significantly faster RTs for the literal interpretation of sentences containing hand-related words compared to the idiomatic interpretation of the same sentences. We interpreted these faster RTs as the product of a facilitation effect. In this same experiment, no significant difference in RTs was found between literal and idiomatic interpretation of sentences containing foot-related words.

Experiment 2 ruled out the possibility that context-sentences alone, in spite of containing action related words, are sufficient to determine motor facilitation. In fact, in this experiment, the same context-sentences, followed by abstract sentences, did not lead to any differential facilitation of the hand response.

In Experiment 3, participants faced the same task as in Experiment 1 but they had to respond with the foot. A facilitation effect was expected during the literal interpretation of sentences containing foot-related words in comparison to the idiomatic interpretation of the same sentences. No significant difference in RTs was expected between the literal and idiomatic interpretation of sentences containing hand-related words. While this latter result was confirmed, our data did not support the former hypothesis. When responding with the foot, participants showed no facilitation effect during the interpretation of foot-related literal sentences.

### Why is the foot different? Or, why is the hand special?

Overall, the data of this study directly support our claim that motor simulation during language comprehension is modulated by the context of utterance only in relation to sentences containing hand-related words. In our study, participants faced a complex pragmatic task while previous studies have been mainly focused on lexical (to understand if a string of letters is a word) or semantic (to make a judgement on the basis of the word meaning) tasks. It was observed that to carry out either a lexical or a semantic task makes a difference in the recruitment of the motor system determining differences in the effects that linguistic processing has on the performance of a concomitant motor act [59]. Also, the extent of the temporal overlapping between linguistic and motor tasks determines another significant difference [10]. It is important to note that we specifically asked the participants of our study to make a judgement of relevancy because we aimed to assess the role of context in the interpretation of target sentences and we needed to be sure that they interpreted target sentences on the basis of context sentences. Previous studies [60]–[61] have already shown differences between task requirements when participants are asked to make meaningfulness judgements, relevancy judgements or when only reading times are measured. The discussion about the methods to be adopted in the research on the mechanisms underlying language processing is still open. The question is which method best explains the processes involved in language comprehension and which is more similar to real-life situations. We believe that findings obtained from different empirical methods, with different tasks requirements, can converge and complement each other to gain a deeper understanding of the processes involved in language comprehension.

In the light of these considerations, we hypothesize that the pragmatic task in our study determined a contextually based interpretation that led to a peculiar modality of recruitment of the motor system. In this case, the recruitment of the motor system could be mediated by activation of prefrontal areas, such as the left dorsolateral prefrontal cortex (DLPF), that have been shown to be involved in idiomatic comprehension and in other linguistic tasks that entail a contextually based semantic disambiguation [62]–[63]. In a top-down process, the DLPF cortex could select the pertinent motor simulation and, also, the adequate motor program to perform the key-press motor act [64]. However, at the behavioural level, the involvement of the sensorimotor system in language comprehension cannot be directly observed except for the comprehension of literal hand-related sentences. Sato et al. [11], [13] suggested that in a semantic task carried out in a delayed condition (when participants perform the key-press motor act 1000 ms after the onset of the linguistic stimulus) no interaction is directly observable between language and the sensorimotor system. Significantly, in our study, RTs were longer than or around 1000 ms in all the conditions. Our peculiar linguistic task very likely determined a “delay-like” condition that made it impossible to directly observe the involvement of the sensorimotor system during language processing except for hand-related action verbs.

Why is the foot different? Or, more precisely, why is the hand special? The intimate relationship between language and hand motor control that has been widely demonstrated [65] could be the reason why, in our task, the involvement of the motor system was evident at the behavioural level only during the comprehension of hand-related literal sentences, although a motor simulation likely also took place in the other conditions. Only further investigations with different techniques (e.g. TMS or neuroimaging) will allow us to fully comprehend this issue.

### Contextual effect on motor simulation

Recently, neuroimaging studies found somatotopic activation of the pre-motor and primary motor cortical areas related to the literal meaning of action verbs during the comprehension of metaphors and idioms [22]–[25]. However, divergent findings have also been obtained in other studies [26], [27], [56]. Although it is not easy to make a direct comparison between these studies because they differ in many respects, such variability in findings suggests that motor simulation during language processing is not constant. It varies under different experimental conditions, also accordingly to the level of interpretation elicited by the task (lexical, semantic, and pragmatic) and the kind of linguistic stimuli used in the experiment. If motor simulation during linguistic comprehension were automatic and invariant, adopting the classic definition of automaticity, namely that a mechanism is considered automatic if it is independent of top-down control [66], we should expect to always find it. Considering that this is not the case, this variability in itself is a challenge to the invariant and non-mediated nature of motor simulation. Lately, contextual effects on motor simulation during linguistic comprehension have been assessed in behavioural [67] and fMRI studies [68]. These findings suggest that contextual information prevails over semantics. However, how precisely this happens is still an open question.

One hypothesis is that motor simulation, in spite of being automatically triggered by intrinsic semantic features, could be inhibited by the processing of contextual information.

Alternatively, context might act before the onset of any motor simulation associated to linguistic processing determining the selection of the contextually salient pattern of motor activation. Interestingly, these mechanisms are not mutually exclusive. Chersi, Ferrari & Fogassi [69] proposed a computational model of neural chains for action in the parietal lobe in which both inhibition and selection mechanisms are involved. According to this model, when contextual cues are enough to understand the agents' intention, the selection of a specific action goal is expressed by the high activity level of a specific neural chain. Instead, when contextual cues are ambiguous, all intentions compatible with the act are prompted and multiple chains are activated in parallel. As soon as more contextual information will become available, non-compatible neural chains will be inhibited. According to this model, motor simulation during linguistic processing can still be considered automatic. Context is a fundamental part of the construction of meaning and can act by selecting the right neural chain of motor simulation, by inhibiting a wrong one or by using both mechanisms at the same time.

## Conclusions

Our data, together with previous findings [48], [50], [67], [68], suggest that motor activation during the processing of action related words is not fixedly associated to the literal meaning of words but depends on the context of utterance. Also, our data support the general claim that automatic mechanisms can be sensitive to the context. This point is particularly important and should be further investigated. Our findings together with previous studies [66] contribute to the discussion on the notion of automaticity. In fact, the classical concept of automaticity is currently under revision and it is now proposed [66] that high-level cognitive mechanisms interacts with automatic processes.

Finally, our data further highlight the intimate relationship between hand and language. Previous studies have widely shown that Broca's area, traditionally considered a language area, is also involved in hand motor control [70], [71]. In fact, Broca's area is both involved in tasks such as complex finger movements, mental imagery of grasping actions, and hand-imitation tasks and in syntactic processing involving reconstruction and interpretation of structured sequences of sentences [70], [72]. No relation like this exists between language areas and areas that control the movements of the foot. Moreover, it has also been suggested that language evolved exactly from manual gestures [65]–[73]. Many reasons have been proposed to support this evolutionary hypothesis and all of them further highlight this intimate relationship between hand and language.
